# Food Outlet Access and the Healthiness of Food Available ‘On-Demand’ via Meal Delivery Apps in New Zealand

**DOI:** 10.3390/nu14204228

**Published:** 2022-10-11

**Authors:** Dru Norriss, Rose Crossin, Angela Curl, Susan Bidwell, Elinor Clark, Tessa Pocock, Ryan Gage, Christina McKerchar

**Affiliations:** 1 Department of Population Health, University of Otago Christchurch, Christchurch 8013, New Zealand; 2 School of Nursing, University of Auckland, Auckland 1023, New Zealand; 3 Department of Public Health, University of Otago Wellington, Wellington 6021, New Zealand

**Keywords:** food, nutrition, demographics, equity, access, online, internet, rapid delivery

## Abstract

Access to unhealthy commodities is a key factor determining consumption, and therefore influences the prevalence of non-communicable diseases. Recently, there has been an increase in the availability of food ‘on-demand’ via meal delivery apps (MDAs). However, the public health and equity impacts of this shift are not yet well understood. This study focused on three MDAs in New Zealand and aimed to answer (1) what is the health profile of the foods being offered on-demand, (2) how many food outlets are available and does this differ by physical access or neighbourhood demographics and (3) does the health profile of foods offered differ by physical access or neighbourhood demographics? A dataset was created by sampling a set of street addresses across a range of demographic variables, and recording the menu items and number of available outlets offered to each address. Machine learning was utilised to evaluate the healthiness of menu items, and we examined if healthiness and the number of available outlets varied by neighbourhood demographics. Over 75% of menu items offered by all MDAs were unhealthy and approximately 30% of all menu items across the three MDAs scored at the lowest level of healthiness. Statistically significant differences by demographics were identified in one of the three MDAs in this study, which suggested that the proportion of unhealthy foods offered was highest in areas with the greatest socioeconomic deprivation and those with a higher proportion of Māori population. Policy and regulatory approaches need to adapt to this novel mode of access to unhealthy foods, to mitigate public health consequences and the effects on population groups already more vulnerable to non-communicable diseases.

## 1. Introduction

Unhealthy commodities, including processed foods that are high in salt, fat and sugar, as well as alcohol and tobacco, are leading risk factors for chronic non-communicable diseases [1]. The burden of non-communicable diseases on health systems globally, including in New Zealand, is substantial, and it is expected to increase dramatically in the next 20 years [2]. Access to these commodities is a key determinant of consumption [3], with increased levels of access to unhealthy food and alcohol outlets associated with poorer health and social outcomes [4,5]. Thus, understanding and managing access to unhealthy commodities is key to minimising harm. 

Over recent years, app-based ‘on-demand’ delivery services for food (both healthy and unhealthy), alcohol and tobacco/nicotine products have emerged. These on-demand services typically coordinate between consumers and multiple sellers, providing a single point of contact and payment. Delivery may then be contracted out to third parties, who collect the food or alcohol from the seller’s premises and deliver it to the consumer within a short time, facilitating immediate consumption. For example, Uber Eats (a meal delivery app; MDA) now connects people with on-demand food from a multitude of restaurants and fast-food outlets in 55 cities and towns across New Zealand [6]. The reach of these services and their uptake is rapidly expanding, both globally and in New Zealand [7,8,9], with demand further accelerated during the COVID-19 lockdowns by the stay home orders and preference for non-contact delivery of goods [10,11]. These on-demand services are distinct from the home delivery of groceries, food boxes or wine orders, which have a longer lead time between order and delivery and are not designed to facilitate immediate consumption. 

Fast-food and alcohol outlets cluster in more deprived communities [12,13,14,15]. There is strong evidence that increased neighbourhood availability of unhealthy food and alcohol is associated with adverse health outcomes, and existing international evidence suggests that similar patterns may be true for on-demand services [16]. While there may be some healthy options offered by MDAs [8], the majority of the food offerings, and those that are most popular, have been shown to be of low or absent nutritional value in studies that covered cities in Australia, England, the Netherlands, the USA and New Zealand [8,16,17,18,19]. On-demand access to unhealthy commodities may therefore present a health risk to those living in more deprived areas, who, in New Zealand, are disproportionally Māori, and who already experience poorer health outcomes [20]. This has the potential to exacerbate existing food access and health inequities that exist in New Zealand [13]. Moreover, regulatory and policy frameworks that seek to control access to unhealthy commodities, if they exist at all, tend to be designed for the built environment rather than the digital environment, and have lagged behind the ability of online platforms (including MDAs) to circumvent those regulations that do exist [21]. 

As yet, however, there is limited research on the health and equity impacts of this rapidly changing unhealthy commodity environment on population health [22,23,24,25]. This study is part of a larger project that seeks to address this knowledge gap specifically in New Zealand. This study focused on the health and equity impacts of MDAs and aimed to answer (1) what is the health profile of the foods being offered on-demand by these MDAs, (2) how many food outlets are available via these MDAs and does this differ by physical access or neighbourhood demographics and (3) does the health profile of foods offered differ by physical access or neighbourhood demographics? By answering these questions, we aim to increase our understanding of the potential health and equity impacts of on-demand access to food via MDAs in New Zealand. 

## 2. Materials and Methods

**Figure 1:**
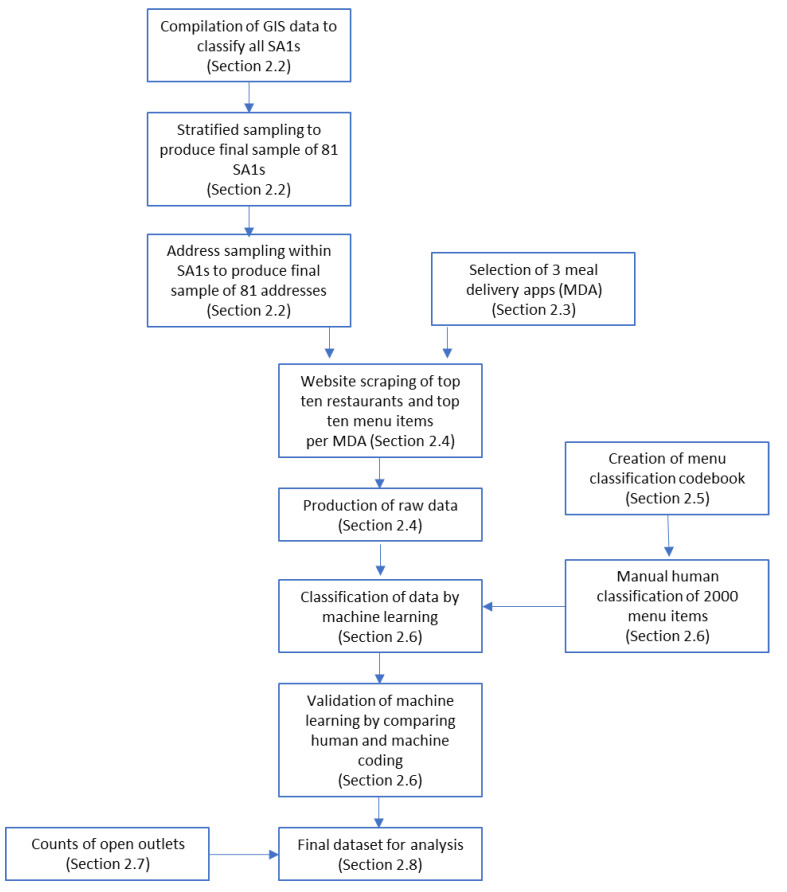
Summary of methods.

To answer these questions, a dataset containing information on the offerings of three MDAs in the three largest cities in New Zealand was created. We first sampled a set of street addresses across a range of demographic variables of interest and recorded the menu items and number of available outlets offered to each address. We then utilised a machine learning approach to evaluate the healthiness of these menu items and examined if the healthiness of items and the number of available outlets varied by the demographic variables of interest. Methods are described in detail below, and summarised as a flowchart in Figure 1. 

### 2.1. Selection of Study Area

The urban areas of Auckland, Wellington and Christchurch are the focus for this study. These areas were chosen as they have relatively established markets for MDAs and a range of on-demand services available [9]. These are the three largest urban centres in New Zealand and thus the study collectively covers approximately 45% of the New Zealand population [26].

### 2.2. Address Sampling

We used a set of representative addresses from Auckland, Wellington and Christchurch to investigate the offerings of the MDAs. Addresses were sampled according to stratification criteria to enable comparisons of MDA offerings by socioeconomic deprivation tertile (i.e., into three groups), density of physical fast-food, takeaway and convenience food tertile and those with a higher proportion of Māori population (defined as Statistical Area 1 (SA1s; a geographic boundary used by Stats NZ) above the 80th percentile of proportion of Māori population in their city). These addresses were used to drive the website scrapers, as virtual naive customers of the MDAs. 

The process was as follows. First, the following data were obtained and imported into ArcGIS Pro (version 2.9.2) [27,28]:

- The feature layer 2018 Census Individual (part 1) total New Zealand by Statistical Area 1 [27] containing geography and census ethnicity data. SA1 is the smallest publicly available, aggregated geographical data output from the New Zealand census.
- The table ‘Statistical Area 1 Higher Geographies 2018’ [28], which contains rural and urban classifications for each SA1, including labelling of Auckland, Wellington and Christchurch.
- NZDep, the standard New Zealand Socioeconomic Deprivation Index by SA1 [29].
- Previously compiled locations of ‘dairy and convenience stores’, ‘fast food’ and ‘takeaways’ from the University of Canterbury GeoHealth Laboratory were combined and added as a feature layer of physical outlets providing a preponderance of unhealthy foods [15].
- Locations of New Zealand street addresses from Toitū Te Whenua Land Information New Zealand (LINZ) [30].

Then, these data were combined and sampled to produce the final list of addresses for the study:

- SA1s were filtered to those of ‘Auckland’, ‘Wellington’ or ‘Christchurch’ in the ‘UR2018_V1_00_NAME’ field of the SA1 higher geographies table.
- SA1s were classified into tertiles of physical access to unhealthy foods. This was done by counting the number of unhealthy outlets within an 800 m buffer of the centroid of each SA1.
- SA1s were classified by NZDep tertile within their respective cities according to their associated NZDep score.
- SA1s were classified into those with a higher proportion of Māori (defined as having a proportion of Māori population in the top quintile among SA1s in each city) or not.

This created a total of 54 groups of SA1s from which to sample: 3 cities × 3 physical access tertiles × 3 NZDep tertiles × 2 ethnicity classifications (3 × 3 × 3 × 2 = 54). Two SA1s were sampled from each city-access-deprivation-other group, and one from each city-access-deprivation-Māori group, creating a final sampled group of 81 SA1s. The LINZ address data [30] were joined to the sampled SA1s. One address was sampled from each SA1, creating the final address list from which searches within the MDAs were performed.

### 2.3. Selection of Meal Delivery Apps (MDAs)

Three MDAs were chosen for investigation. The first two, Uber Eats and Menulog (part of Just Eat Takeaway), are the first and second market leaders in New Zealand [7], and both part of multinational publicly traded companies. By contrast, the third MDA, delivereasy, is currently a New Zealand-based and privately held company. This selection enables a broad view of the New Zealand market, as well as comparison between domestic and international MDAs. All MDAs are active in the cities chosen for study.

### 2.4. Data Collection—Website Scraping

The goal was to simulate a naive user purchasing food or drink at 6 p.m. on a Tuesday, Wednesday or Thursday. Collection was done as close to this time as possible to give the most accurate representation of what the selected MDAs offered. The desktop browser version of the three different companies’ apps (Uber Eats, Menulog and delivereasy) was used for consistency. Website scraping (automated collection of data from MDAs) was performed by automated computer scripts written in the Python scripting language, utilising Selenium WebDriver [31]. In general, the automated process was the same across the three services investigated, and replicated the steps that a human user of the website would perform:

- Clear all browsing data and reset the test browser to ensure that any previous searches do not influence future results;
- Search the address of interest;
- Set time for delivery (if supported by the site; searches were performed as close to the time of interest as possible);
- Select each of the first 10 food outlets shown;
- For each food outlet, select the first 10 items;
- Record the item name and description for each item.

This meant that a maximum of 8100 items per MDA could be recorded if all addresses were available, had no errors and had at least 10 restaurants available and 10 items available per restaurant (81 × 10 × 10 = 8100). Screenshots were automatically taken and saved at the address, food outlet and item level, enabling manual verification of the data.

Defining the ‘first 10’ food outlets and restaurants displayed in the MDAs was not straightforward, owing to the differences in design, layout and functionality between sites. For this project, the top 10 were considered to be the first 10 items seen when scanning the page from left to right, top to bottom, including scrolling of the page if necessary, but not from interacting with sub-elements within the page (e.g., a ‘carousel’ view). Examples from each service, with the numbering of food outlets and menu items, are shown in Figure S1. All pages were viewed in the Firefox web browser (version 91.7.1esr) on Windows 10, at a desktop resolution of 1920 × 1080, with no display scaling.

For Uber Eats, data were collected on Tuesday–Thursday, 10–12 May 2022, between 12:30 p.m. and 5:14 p.m. A delivery time of 6 p.m. was selected on the Uber Eats interface. For Menulog, data were collected on Wednesday–Thursday, 4–5 May, between 5:00 p.m. and 7:08 p.m. As there was no way to select a delivery time without creating an account and logging in, data collection was approximately centred around 6 p.m. For delivereasy, data were collected on 5 May 2022, between 4:00 p.m. and 5:12 p.m. A delivery time of 6 p.m. was selected. These parameters were selected to represent time periods in which it could be considered ‘typical’ to order a meal (i.e., a weeknight dinner time). 

### 2.5. Classification of Food Items

Classifying the ‘healthiness’ of food items based on only an item name and description, some of which can be very brief, is challenging, and there is no universally established or agreed process. Partridge et al. [19], in a similar recent investigation, took a pragmatic approach, where they human-classified popular items on Uber Eats as either ‘core’ or ‘discretionary’ based on the Australian Dietary Guidelines [32]. Recognising the complexity and diversity of offerings available in our dataset (from tea bags, to quail eggs, to burger meals), we built on this approach by separately coding items for ‘core’ and ‘discretionary’ components. With the input of two university-trained nutritionists (R.G. and C.M.), a classification manual was developed [33] with reference to the Eating and Activity Guidelines for New Zealand Adults [34]. From these guidelines, a series of item properties were derived (from ‘Eating Statements’ 1 and 2) to inform a core score (‘Variety’, ‘Fruit/Veg’, ‘Whole Grains’, ‘Dairy’, ‘Protein’), and a discretionary score (‘Saturated Fat’, ‘Salt’, ‘Sugar’, ‘Processed’). 

Each component was scored as either 0, 1 or 2, corresponding to its healthiness (or unhealthiness) when compared against the New Zealand nutrition guidelines. For example, sugary drinks were assigned a score of 2 because guidelines recommend choosing drinks with little or no added sugar. An overall score was obtained by subtracting the discretionary score from the core score, and ranged from −2 (least healthy) to 2 (most healthy). Preserving the separate components in this way allowed for later analysis by either overall score, core score or discretionary score. 

For practicality purposes and to facilitate consistency of the manual classification of menu items, 36 intermediate categories were developed. These represented menu items or types of menu items often seen in the dataset, and emerged from early attempts by the team to code the diverse and complex dataset. Examples of these categories include ‘Salad (+ or − dressing or meat)’, with a score of 2 core and 0 discretionary; ‘Burger only—premium’, with a score of 2 core and 2 discretionary; and ‘Pizza meal deal’, with a score of 0 core and 2 discretionary.

All alcoholic drinks were coded as 0 core and 2 discretionary (giving an overall score of −2). Specific consideration of alcohol available through MDAs is the subject of a future article by this project team; as the focus of this article was food, no separate analysis of alcohol was done. At the time of data collection, Uber Eats and delivereasy both offered alcohol; Menulog did not.

To allow for simple comparisons of proportions, a binary classification of foods as ‘healthy’ or ‘unhealthy’ was assigned based on their final ‘score’ (core minus discretionary), with scores of 1 and 2 being considered ‘healthy’, and scores of −2, −1 and 0 ‘unhealthy’.

### 2.6. Machine Learning for Classification of Full Dataset

‘Machine learning’ is a general term for a set of techniques and processes whereby a computer system analyses data to detect patterns and predict outcomes [35]. This can have considerable advantages over standard programmatic techniques to organise data, whereby the patterns must be known and described in advance in such a way as to be programmable. In the case of the present study, for example, this might involve creating a hyper-granular scoring system to identify and weight ‘fry’, ‘fries’, ‘fried’ while also considering the context for whether ‘chicken’ or ‘chips’ are also present, to be able to distinguish and score stir-fried chicken from potato fries. The complexity of defining this programmatically may rapidly consume resources beyond manually categorising ~20,000 items, as resource-intensive as this already is.

As an alternative, we used a supervised machine learning technique to ‘train’ a model to code the full dataset. The process was as follows: following collection of data (over 20,000 menu items), a random sample of 2000 items was categorised manually (by D.N.) according to the classification manual [33]. These classified data were then processed with a Python script using a multi-layer perceptron neural network [36]. The output was a spreadsheet with a full set of classified menu items, which were used for the remaining analysis.

Validation was carried out using k-fold cross-validation [37]. This is a procedure whereby a portion of the human-classified data are held back from the training algorithm, allowing the performance of the model to be evaluated on data to which it is naive. The ‘folds’ are created by randomly splitting the training data into subsets (in this case, 5) of equal size, from which multiple tests of the model are run by holding back a different subset of training data each time. Therefore, all of the training data are still used to evaluate the model. The average performance across these tests gives an estimate of the likely performance of the model on new data. In this case, the model scored 88.8% for ‘core’ scores (that is, the model predicted the same score as the human scoring (D.N.) for ‘core’ components 88.8% of the time) and 88.2% for ‘discretionary’ components.

To compare the performance of the machine learning with the alternative of manual classification by a team, two researchers (T.P., E.C.) independently classified 500 items from the training data. Agreement for ‘core’ scores was 81.2% and 80.4% as compared to the training data, and 84.6% and 86.6% for ‘discretionary’ scores, respectively. Given that the machine learning scores surpassed the manual scores, it shows that, in this situation, machine learning performed better at the task of categorising menu items to imitate the training data (coded by D.N.) than the researchers using the classification manual. It is possible that with further cycles of categorisation, manual review and consensus discussion between researchers, the machine learning performance could have been approached or surpassed. However, given the large dataset and good performance of the machine learning categorisation, we judged this as a robust approach.

### 2.7. Counting Open Outlets

As well as gathering data about menu items, scripts were used to count the number of open outlets available at each address from each MDA. These data were captured on Tuesday–Thursday, 21–23 June 2022. Data were collected as close to 6 p.m. as possible, and for those MDAs that supported it (Uber Eats and delivereasy), a delivery time of 6 p.m. was selected. 

### 2.8. Analysis

Numbers and percentages of menu items for each score (−2, −1, 0, 1, 2) were counted and calculated for each MDA. 

Chi^2^ tests were performed for each MDA to evaluate the relationship between proportions of ‘healthy’ and ‘unhealthy’ foods and the three factors of NZDep 2018 scores, physical density of junk-food outlets and Māori population density. *p*-values < 0.05 were considered statistically significant.

For the counts of available outlets, the median, first quartile and third quartile were calculated for each grouping of addresses according to the three stratification factors. 

## 3. Results

Table 1 shows proportions of items by ‘healthiness’ score for each MDA. Data were obtained for Uber Eats and Menulog from all 81 addresses, while, for delivereasy, 59 addresses were successful. The remaining 22 addresses were unavailable due to lack of service coverage; 19 of these were Auckland addresses. 

All MDAs showed a strong bias towards lower-scoring (‘unhealthy’) items. When considering the total of unhealthy items as those scoring zero or less, the total percentage of unhealthy items was 77.1% from Uber Eats, 81.2% from Menulog and 72.8% from delivereasy. Although there were some large proportional differences in score categories (e.g., delivereasy has approximately 2–3 times the proportion of items scoring 2 as compared to the other two MDAs), the greatest absolute difference is 8.7% between items scoring 0 from Menulog and delivereasy. This indicates that in terms of our scoring system of ‘healthiness’, these three MDAs are more alike than they are different.

Table 2 shows the median number (with first and third quartiles) of available outlets to purchase food from for each MDA by the three demographic variables of interest (NZDep 2018, physical outlet density and proportion of Māori population). Data were obtained without errors for Uber Eats, Menulog and delivereasy for 80, 81 and 59 addresses, respectively. The one address unavailable for Uber Eats was due to a persistent page-loading failure, preventing automated collection. The 22 addresses unavailable for delivereasy were due to lack of service coverage; 18 of these were Auckland addresses. 

Overall, there was a large difference in the median number of outlets offered by the three MDAs. Delivereasy had the fewest outlets (55), with Menulog almost doubling (95) and Uber Eats tripling (156.5) that number. 

No clear or linear pattern was apparent between the median number of available outlets and NZDep 2018 for any of the three MDAs. In contrast, addresses from areas with a higher population proportion of Māori had approximately two thirds of the available outlets when compared with other areas for all three MDAs.

When considering the number of outlets available digitally on MDAs, compared to physical outlet density, both Uber Eats and Menulog showed increased digital outlet availability for addresses that had the highest density of physical outlets. However, this was not observed with delivereasy (similar level of digital outlet availability across physical density tertiles). 

Table 3 shows proportions of menu items scoring 1 or 2 (‘healthy’), and those scoring 0, −1 or −2 (‘unhealthy’), for each of the three MDAs. Results are presented as proportions by each MDA and separately for each of the three demographic factors of interest (NZDep 2018, physical outlet density and proportion of Māori population), resulting in nine (3 × 3) sub-tables. The Chi^2^ test statistics and *p*-values are displayed below each of the nine sub-tables.

Statistically significant differences in the proportions of ‘healthy’ and ‘unhealthy’ menu items offered by Menulog were found for all three demographic factors: NZDep2018 (Chi^2^ = 20.9341, *p*-value < 0.0001), physical fast-food outlet density (Chi^2^ = 13.905, *p*-value = 0.0010) and Māori population (Chi^2^ = 12.7487, *p*-value = 0.0004). The results show that for Menulog, a greater proportion of unhealthy food was offered at addresses that were more socioeconomically deprived, had lower physical fast-food outlet density and had a higher proportion of Māori population. No statistically significant differences were seen among demographic or physical outlet density for menu items offered by Uber Eats or delivereasy.

## 4. Discussion

This study aimed to characterise and quantify the healthiness of menu items offered by MDAs in New Zealand, and to determine if the number of food outlets available via MDAs and the health profile of the foods offered differed by physical access or neighbourhood demographics. By answering these questions, we aimed to increase our understanding of the potential health and equity impacts of on-demand access to food in the three most populous cities in New Zealand. We characterised the first 10 menu items from the first 10 food outlets offered to naive users of three popular MDAs in New Zealand (Uber Eats, Menulog, delivereasy), and stratified results according to socioeconomic deprivation, density of physical takeaway and convenience food outlets and proportion of Māori residents. To the best of our knowledge, this is the first characterisation of on-demand food in New Zealand outside of Auckland, and it extends previous studies by comparing three MDAs.

The overall picture is one of uniformity between MDAs and of menu offerings across the variables of interest. Importantly, more than three quarters of menu items offered by all services were unhealthy (score of ≤ 0), and approximately 30% of all menu items across the three MDAs scored at the lowest level of healthiness (score of −2). This is consistent with findings from international research. A study that sampled meal delivery options across 10 locations in each of Melbourne, Amsterdam and Chicago [16] found that most advertised food types were considered unhealthy. Similarly, almost 70% of food items offered by delivery apps in a Brazilian metropolis were found to be ultra-processed, ready-to-eat meals [38], while Australian researchers [19,39] classified over 80% of all menu items offered by online food delivery platforms as discretionary.

By considering the healthiness of menu items by demographics, we sought to understand whether inequities in the healthiness of menu items were evident in New Zealand. Statistically significant differences by demographics were identified in one of the three MDAs in this study, which suggested that the proportion of unhealthy food offered was highest in areas with greatest socioeconomic deprivation and those with a higher proportion of Māori population. However, it is important to note that the largest absolute difference between any groups compared was a 4.2% difference in items scoring ≤0 between the least deprived and most deprived tertiles for Menulog. This is a difference of less than a single item, on average, in the first 10 items displayed to a user. When considering our demographic stratification, our results show that the majority of menu items (over 70% in all cases) are unhealthy across the three MDAs.

These MDAs represent an increase in access to disproportionally unhealthy food for all residents of Auckland, Wellington and Christchurch. While any associated change in consumption cannot be known from this study, it is reasonable to conclude that these services are likely to drive the consumption of unhealthy foods [40]. Although we did not observe consistent increases in unhealthy food offerings in areas of low socioeconomic status or high Māori population, nevertheless, on-demand access to unhealthy foods may interact with different population groups in different ways. For example, increases in access via MDAs for affluent population groups, with high nutrition literacy, and the availability of healthy food, may have a different effect compared to an increase in access for populations living in areas where the availability of healthy food is already limited, and existing food access inequities could be compounded.

On-demand food delivery exists within a wider food environment that, in New Zealand, is characterised by heavy marketing and the availability of unhealthy foods across a range of domains [13]. For example, in New Zealand supermarkets, 69% of packaged foods available are ultra-processed and, in their marketing, unhealthy foods are promoted over fresh fruit and vegetables at a ratio of 4 to 1 [13]. Over the COVID-19 lockdown period in 2020, online food delivery by supermarkets became increasingly used by consumers. Although there was an increase in home-cooked meals over lockdown, there was also an increase in snacking on high-sugar and high-sodium foods [41]. While online supermarket shopping does not use techniques such as shelf placement or end-of-aisle displays to market foods, they do instead focus on which product is presented first on a screen [42]. The COVID-19 lockdowns were also used by food companies, especially fast-food companies, as an opportunity to leverage the pandemic and people’s vulnerabilities as an opportunity to market unhealthy foods [43]. It is likely that this trend towards online food and meal delivery will continue post-pandemic. Despite these issues, the potential exists for the digital environment to ‘nudge’ consumers towards healthy food purchases, and future research should focus on how this could be operationalised in MDAs [44].

Work to consider how these valuable and sensitive (commercially and politically) data should be made easily accessible to those who generate them in the first place, and to those who work on behalf of all to protect and promote health and wellbeing, is essential as these types of services proliferate across all areas of life. This raises questions around data sovereignty [45,46,47]. Granular and powerful data are generated by individuals using on-demand services, which may be then collected and used to improve the performance of the MDAs with respect to business goals. This may include, for example, finding patterns in use across populations and running trials on when and how to prompt and offer different outlets and items to trigger further and higher-value purchases. These goals are opaque to individual users of the services, and may be at odds with public health goals [48,49]. An Australian initiative where researchers partnered with young ‘citizen scientists’ to track the promotion of unhealthy food, alcohol and gambling that they saw on their social media feeds is an example of efforts to expose the ‘dark marketing’ strategies that target consumers [50].

Our study has several limitations that must be acknowledged. Examining which menu items are presented to a naive user was a necessary part of the study design. However, this is also a limitation, as, by definition, any repeat user of an MDA will no longer be naive. After creating an account and ordering, it is likely that food outlets and menu items presented to the user will be optimised based on their previous orders and other information provided on sign-up [40,50,51]. Over time, these will be much more significant factors for the potential consumption and therefore health impacts of these services than what they show to a new user. Exploring this issue further is a logical next research step.

The observed pattern on Menulog of a greater number of unhealthy foods accessible to addresses with a higher proportion of Māori populations should be interpreted cautiously. It is possible that the stratification strategy has too aggressively selected some SA1s, magnifying unknown confounders. For example, only three SA1s (from which one was sampled, and an address then sampled) remained after stratifying for Christchurch, NZDep Tertile 1, outlet density Tertile 3 and a high proportion of Māori residents. Furthermore, delivereasy has an incomplete presence in Auckland (as observed by the lower number of addresses successfully sampled), and the sequencing of their rollout may skew these results.

Our strategy of sampling from the three major urban centres excludes the possibility of comparison between large urban areas and smaller towns or rural areas, though any such comparison would be difficult to interpret as services are less mature outside of main centres. We acknowledge, however, that this may be a source of confounding, particularly given that rural areas have a higher proportion of the Māori population in New Zealand [52].

Using the desktop browser versions (as opposed to mobile web or native mobile applications) of these MDAs has an unknown effect on the results. It is unknown what proportion of users order from smartphone apps as compared to the desktop versions. However, smartphone apps are more difficult to scrape data from, and were therefore not feasible for our study design.

Finally, the use of our own novel classification system for food items limits comparison with other studies. Although our classification is based on evidence-based guidelines and discussion with university-trained nutritionists, it is not itself validated. Further work to standardise the categorisation and evaluation of the ‘healthiness’ of foods based on incomplete or limited information, allowing rapid computerised and reproducible research on large datasets, would benefit future research of MDAs. We also acknowledge that our machine learning method did not achieve 100% accuracy, but note that the accuracy of the machine learning exceeded that of human coders. Similar research has, however, used a combination of validated expert opinion and nutritional guidelines to classify foods and outlets as healthy or unhealthy based on item names and descriptions [19].

Our study adds to the body of evidence examining the complex nexus created by high levels of non-communicable diseases and the opaque influence of unregulated MDAs. A subsequent paper from our wider study will complement this research by examining issues around app-based on-demand alcohol delivery. Researchers and policy makers across multiple jurisdictions are grappling with similar challenges posed by the increasing uptake of MDAs and growing evidence of their negative health implications [23,40,53,54,55]. Addressing these challenges requires a ‘multisector and systemic approach’ [54] to create structural change in the overall food environment. Interventions that target the market environment have been shown to be more effective than those that simply support informed choice [40]. A national food and nutrition strategy, such as implemented in Scandinavian countries [56,57], provides the framework to bring together government regulators, researchers, policy makers and public health experts in an overarching, collaborative effort. Further evidence that considers the digital food environment is needed to underpin the strategy and develop effective policies in a range of different areas, such adapting existing laws to the digital environment, monitoring the influence of marketing strategies, promoting healthy food through clear public health messages and incentivising food providers to deliver healthier meal options [40,54]. Ultimately, MDAs might then, as the WHO has suggested, be used as ‘a driving force to improve diets and reduce non-communicable disease’ [40] (p. 26).

## 5. Conclusions

Over three quarters of the first 10 menu items offered by the first 10 food outlets on Uber Eats, Menulog and delivereasy MDAs are unhealthy, among a sample of addresses in Auckland, Wellington and Christchurch, New Zealand. While statistically significant differences in the proportion of unhealthy foods by NZDep 2018 tertiles, the physical density of takeaway and convenience stores and addresses with and without a higher proportion of Māori population were observed for one MDA, these differences were relatively small (one menu item in ten difference). The impact of such differences on consumption is currently unknown. One limitation of our research was that it assumed a ‘naïve’ user, i.e., search results were not guided by previous purchasing behaviour. Future research could extend our findings by considering how the healthiness of menu items differs based on prior purchasing or demographics. Furthermore, it will be important for future research to consider how different menu items are marketed through MDAs, and whether this influences purchasing behaviour. MDAs represent a novel mode of enhancing access to unhealthy food. As such, MDAs may have negative public health consequences and a compounding effect for population groups already more vulnerable to non-communicable diseases and the consumption of unhealthy foods. It is vital that policy environments keep pace with technological change, so that MDAs can be effectively regulated.

## Figures and Tables

**Table 1:** Summary of menu items (maximum first 10 items from first 10 outlets) by healthiness score per meal delivery app.

	Items by Score, *n* (%)					
	Unhealthy −2	−1	0	1	Healthy 2	Total
**Uber Eats**	2361 (31.7)	2277 (30.5)	1114 (14.9)	1568 (21.0)	138 (1.9)	7458 (100.0)
**Menulog**	2197 (27.2)	2681 (33.2)	1680 (20.8)	1340 (16.6)	178 (2.2)	8076 (100.0)
**delivereasy**	1609 (30.1)	1632 (30.6)	646 (12.1)	1134 (21.2)	316 (5.9)	5337 (100.0)

Note—the total number of items for each MDA, from a maximum possible number of 8100, was limited by service address availability, whether there were ten restaurants available at each address and whether there were ten menu items available within the top ten restaurants.

**Table 2:** Available outlets by NZDep, physical outlet density and Māori population. Median (first quartile, third quartile).

		**Uber Eats**				**Menulog**				**Delivereasy**			
		**Tertile (1 = Least Deprived)**				**Tertile (1 = Least Deprived)**				**Tertile (1 = Least Deprived)**			
		**1**	**2**	**3**	**Overall**	**1**	**2**	**3**	**Overall**	**1**	**2**	**3**	**Overall**
NZDep 2018	Outlets	181 (71.5, 258)	143 (85.5, 187.5)	163 (107, 202.5)	156.5 (87.5, 209.5)	113 (19, 162)	75 (18, 149.5)	101 (14, 146)	95 (16, 158)	51 (31, 91.5)	71.5 (53.5, 93.5)	48 (31.5, 92.5)	55 (33.5, 93.5)
		**Tertile (1 = Least Dense)**				**Tertile (1 = Least Dense)**				**Tertile (1 = Least Dense)**			
		**1**	**2**	**3**	**Overall**	**1**	**2**	**3**	**Overall**	**1**	**2**	**3**	**Overall**
Physical Density	Outlets	119 (82.5, 190.5)	126 (76.5, 187)	185.5 (153.25, 280)	156.5 (87.5, 209.5)	76 (13, 119.5)	82 (10, 142.5)	148 (21, 170.5)	95 (16, 158)	53 (33, 92)	48 (32, 89)	56 (41.5, 95)	55 (33.5, 93.5)
		**Higher Proportion Māori**		**Other**	**Overall**	**Higher Proportion Māori**		**Other**	**Overall**	**Higher Proportion Māori**		**Other**	**Overall**
Māori Population	Outlets	119 (85, 182.5)		170 (102, 270)	156.5 (87.5, 209.5)	75 (22, 119)		105.5 (13.25, 163.75)	95 (16, 158)	34 (18, 87)		55 (44.5, 94)	55 (33.5, 93.5)

Note—outlets are presented as the median (first and third quartile).

**Table 3:** Proportions of menu items by NZDep, physical outlet density and Māori population. N items scoring (%).

		**Uber Eats**				**Menulog**				**Delivereasy**			
		**Tertile (1 = Least Deprived)**				**Tertile (1 = Least Deprived)**				**Tertile (1 = Least Deprived)**			
		**1**	**2**	**3**	**Total**	**1**	**2**	**3**	**Total**	**1**	**2**	**3**	**Total**
NZDep 2018	‘Healthy’ ^1^	543 (21.7)	607 (24.2)	556 (22.8)	1706 (22.9)	545 (20.2)	542 (20.2)	431 (16.0)	1518 (18.8)	501 (26.9)	468 (28.6)	481 (26.2)	1450 (27.2)
	‘Unhealthy’ ^2^	1965 (78.3)	1905 (75.8)	1882 (77.2)	5752 (77.1)	2150 (79.8)	2143 (79.8)	2265 (84.0)	6558 (81.2)	1362 (73.1)	1170 (71.4)	1355 (73.8)	3887 (72.8)
	Total Items	2508 (100)	2512 (100)	2438 (100)	7458 (100)	2695 (100)	2685 (100)	2696 (100)	8076 (100)	1863 (100)	1638 (100)	1836 (100)	5337 (100)
		**Chi^2^ = 4.5033, *p*-Value = 0.1052**				**Chi^2^ = 20.9341, *p*-Value < 0.0001**				**Chi^2^ = 2.5747, *p*-Value = 0.2760**			
		**Tertile (1 = Least Dense)**				**Tertile (1 = Least Dense)**				**Tertile (1 = Least Dense)**			
		1	2	3	Total	1	2	3	Total	1	2	3	Total
Physical Density	‘Healthy’	623 (24.4)	539 (22.3)	544 (21.8)	1706 (22.9)	490 (18.2)	462 (17.2)	566 (21.0)	1518 (18.8)	405 (27.1)	473 (26.9)	572 (27.4)	1450 (27.2)
	‘Unhealthy’	1928 (75.6)	1875 (77.7)	1949 (78.2)	5752 (77.1)	2198 (81.8)	2231 (82.8)	2129 (79.0)	6558 (81.2)	1088 (72.9)	1283 (73.1)	1516 (72.6)	3887 (72.8)
	Total Items	2551 (100)	2414 (100)	2493 (100)	7458 (100)	2688 (100)	2693 (100)	2695 (100)	8076 (100)	1493 (100)	1756 (100)	2088 (100)	5337 (100)
		**Chi^2^ = 5.4384, *p*-Value = 0.0659**				**Chi^2^ = 13.905, *p*-Value = 0.0010**				**Chi^2^ = 0.9497, *p*-Value = 0.5409**			
		**Higher Proportion Māori**		**Other**	**Total**	**Higher Proportion Māori**		**Other**	**Total**	**Higher Proportion Māori**		**Other**	**Total**
Māori Population	‘Healthy’	566 (23.1)		1140 (22.8)	1706 (22.9)	448 (16.6)		1070 (19.9)	1518 (18.8)	459 (27.2)		991 (27.1)	1450 (27.2)
	‘Unhealthy’	1888 (76.9)		3864 (77.2)	5752 (77.1)	2250 (83.4)		4308 (80.1)	6558 (81.2)	1227 (72.8)		2660 (72.9)	3887 (72.8)
	Total Items	2454 (100)		5004 (100)	7458 (100)	2698 (100)		5378 (100)	8076 (100)	1686 (100)		3651 (100)	5337 (100)
		Chi^2^ = 0.0745, *p*-value = 0.7848				Chi^2^ = 12.7487, *p*-value = 0.0004				Chi^2^ = 0.0038, *p*-value = 0.9507			

^1^ ‘Healthy’ is the total number of items scoring 1 or 2. ^2^ ‘Unhealthy’ is the total number of items scoring 0, −1 or −2.

## Data Availability

The coding manual used to determine the healthiness of menu items has been uploaded and is available at (https://www.otago.ac.nz/christchurch/research/populationhealth, accessed on 11 September 2022).

## Supplementary Materials

The following supporting information can be downloaded at: https://www.mdpi.com/article/10.3390/nu14204228/s1, Figure S1: Counting Top 10 Food Outlets and Menu Items.
