# Supplementary material for: Food Outlet Access and the Healthiness of Food Available ‘On-Demand’ via Meal Delivery Apps in New Zealand
Source: Nutrients. 2022 Oct 11;14(20):4228. doi: 10.3390/nu14204228 (PMC9607030; doi:10.3390/nu14204228)

Supplementary Material 1: Counting Top 10 Food Outlets and Menu Items

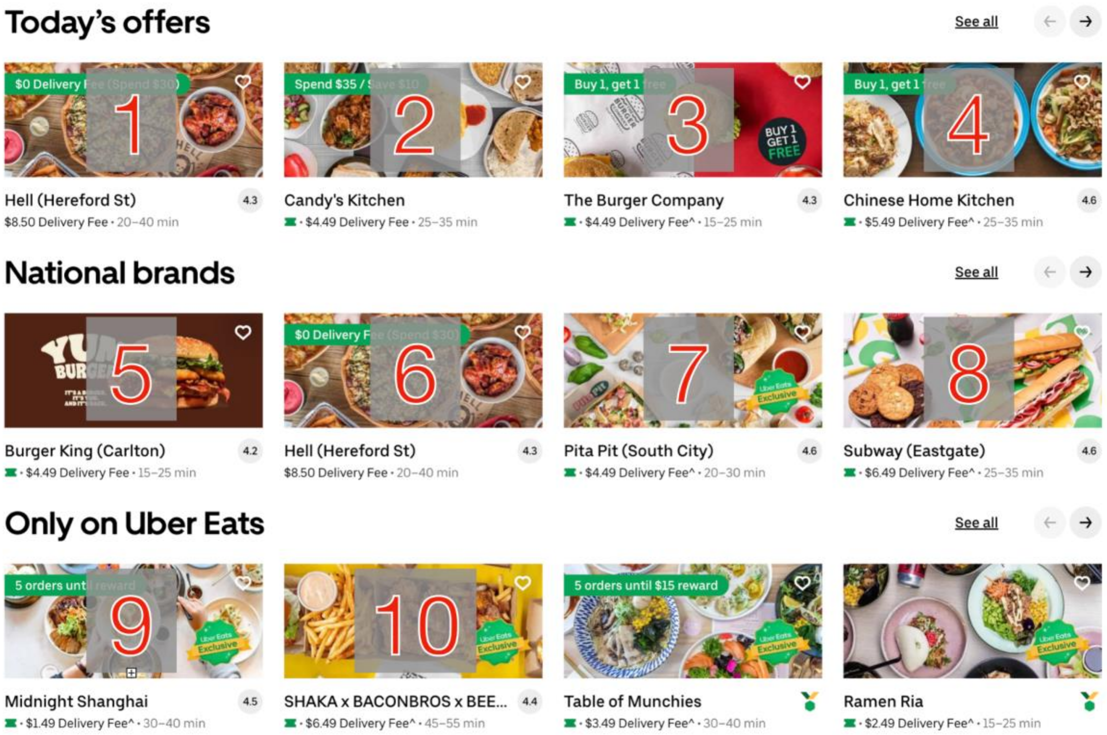

Uber Eats Food Outlets Example

Note that even though each row can be scrolled with the arrows to the right of the "See all" link, food outlets not visible without interacting with the page are not counted.

Uber Eats Items Example

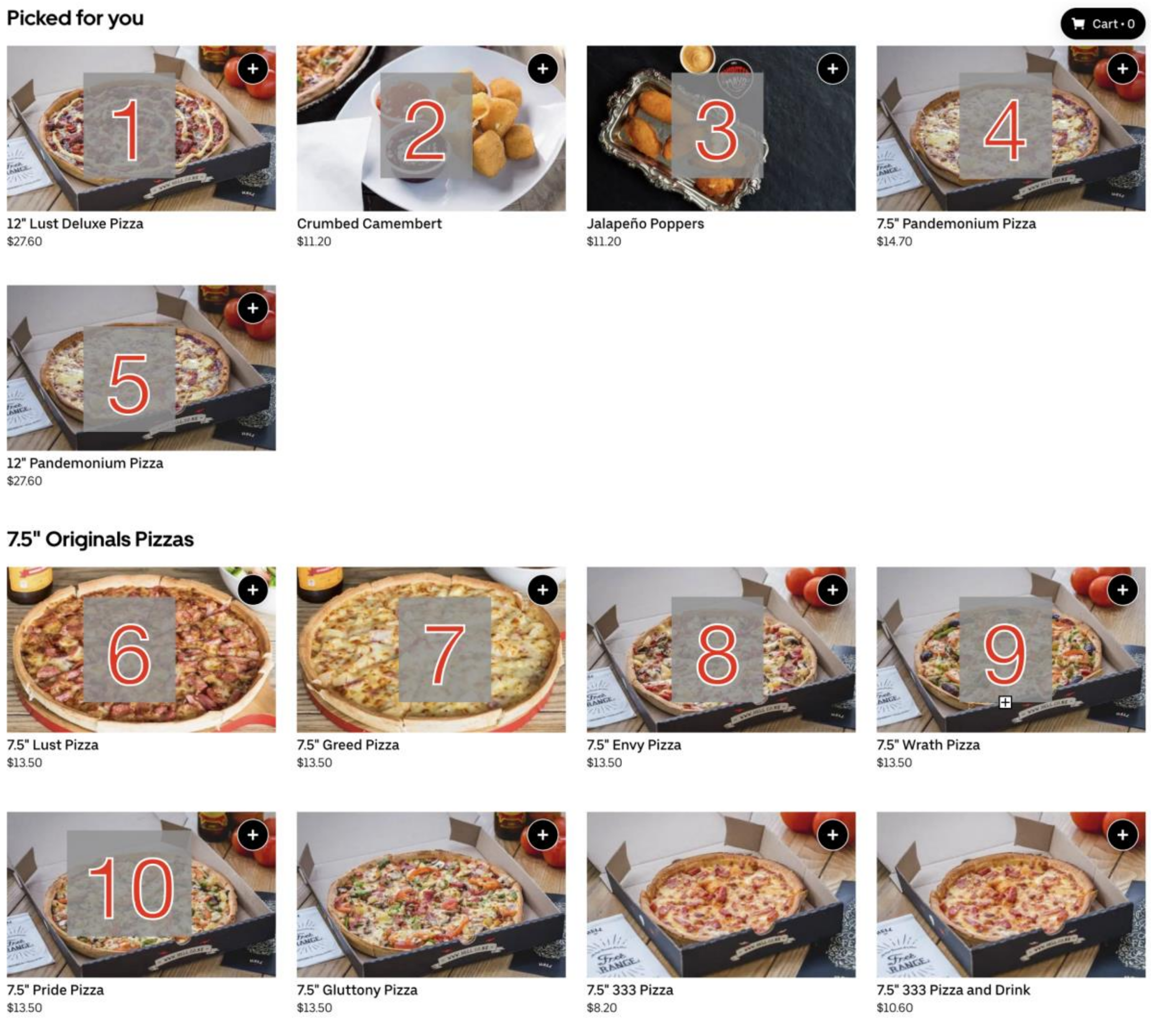

In contrast to the food outlets page, there are no horizontally scrollable subsections, making the selection of the top 10 more straightforward.

Menulog Food Outlets Example

First view:

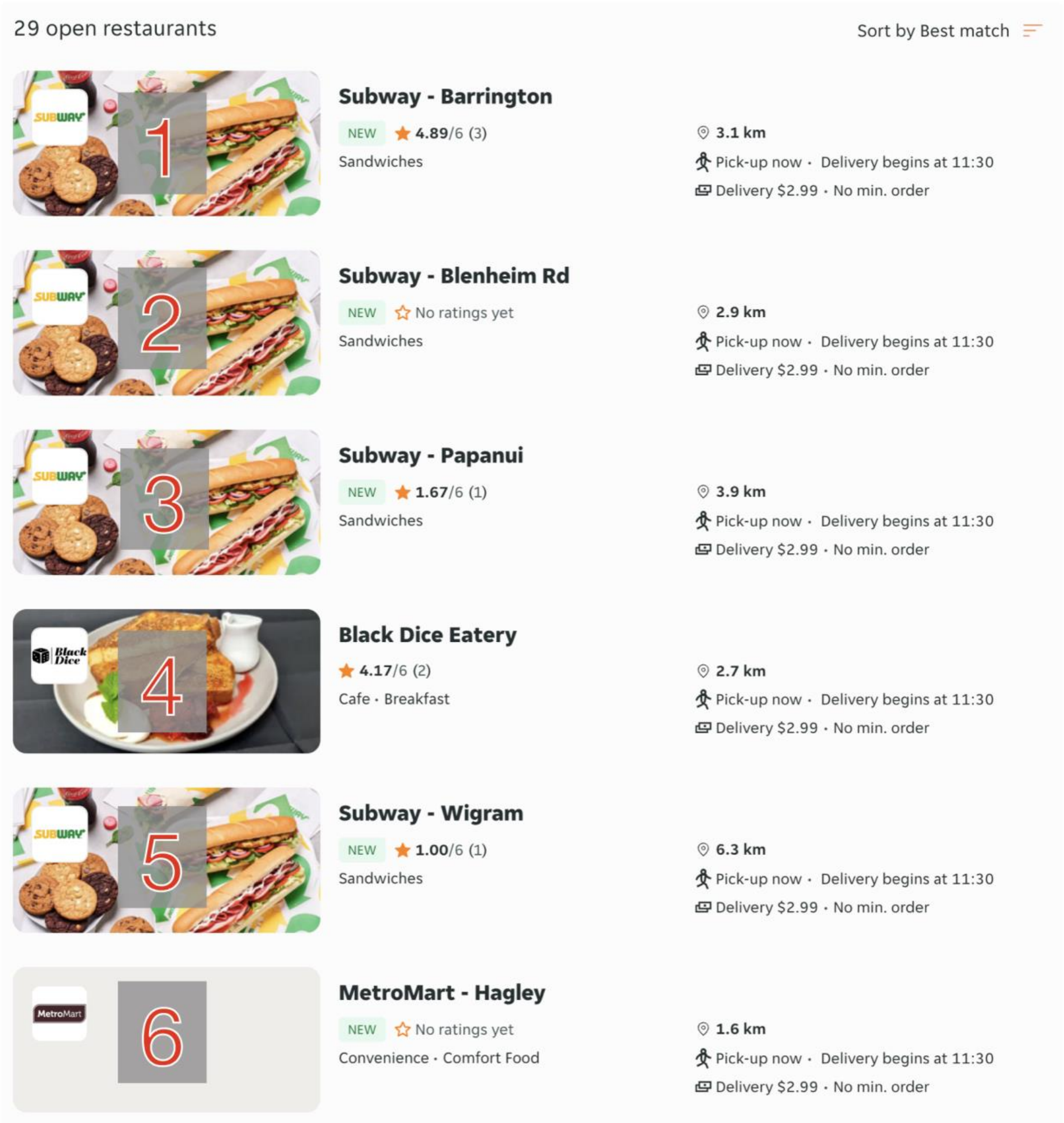

Menulog Food Outlets Example (continued)

After page scroll:

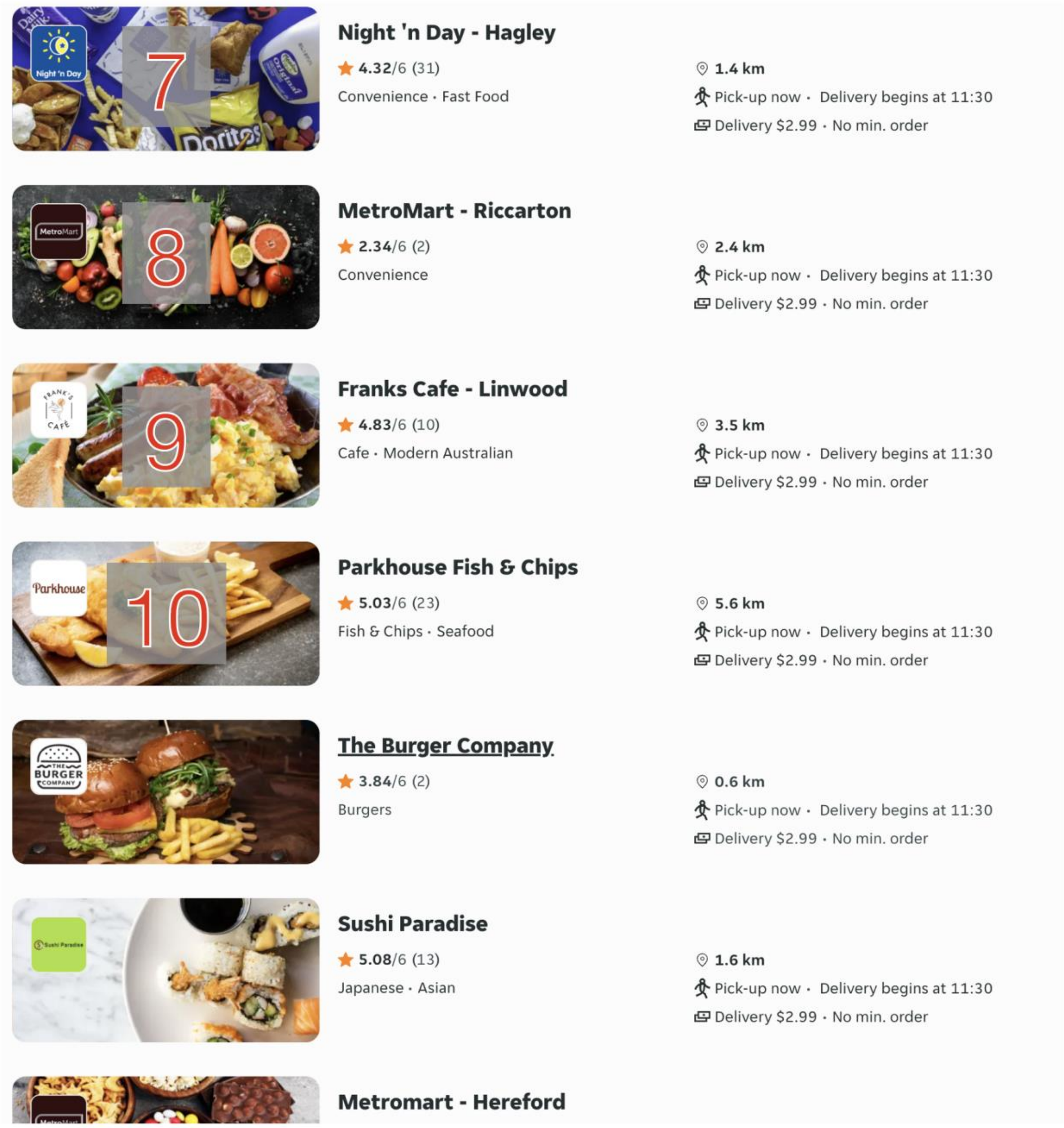

Menulog Menu Items Example

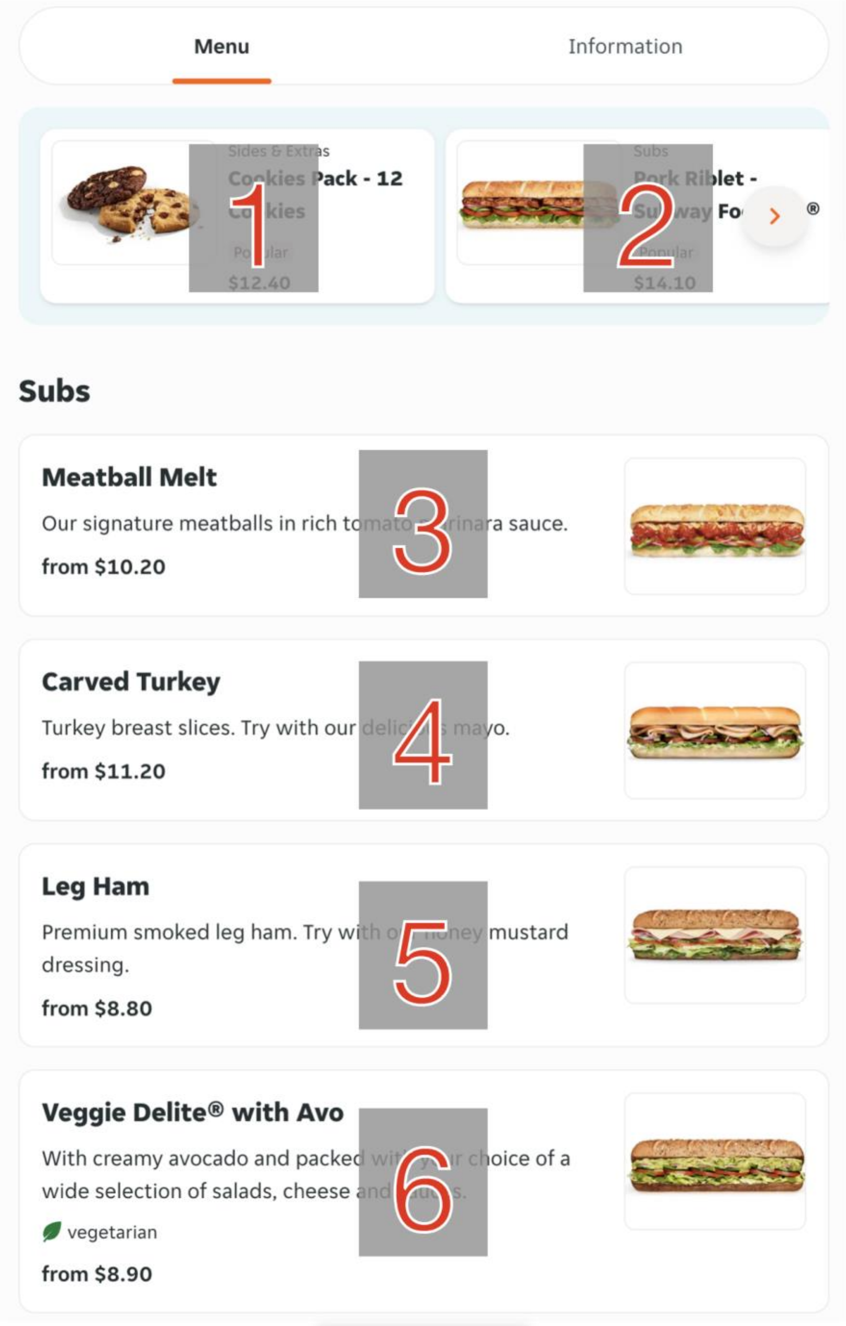

The first two items are those visible in the scrollable carousel highlighted at the top of the menu.

Menulog Menu Items Example (continued)

After page scroll:

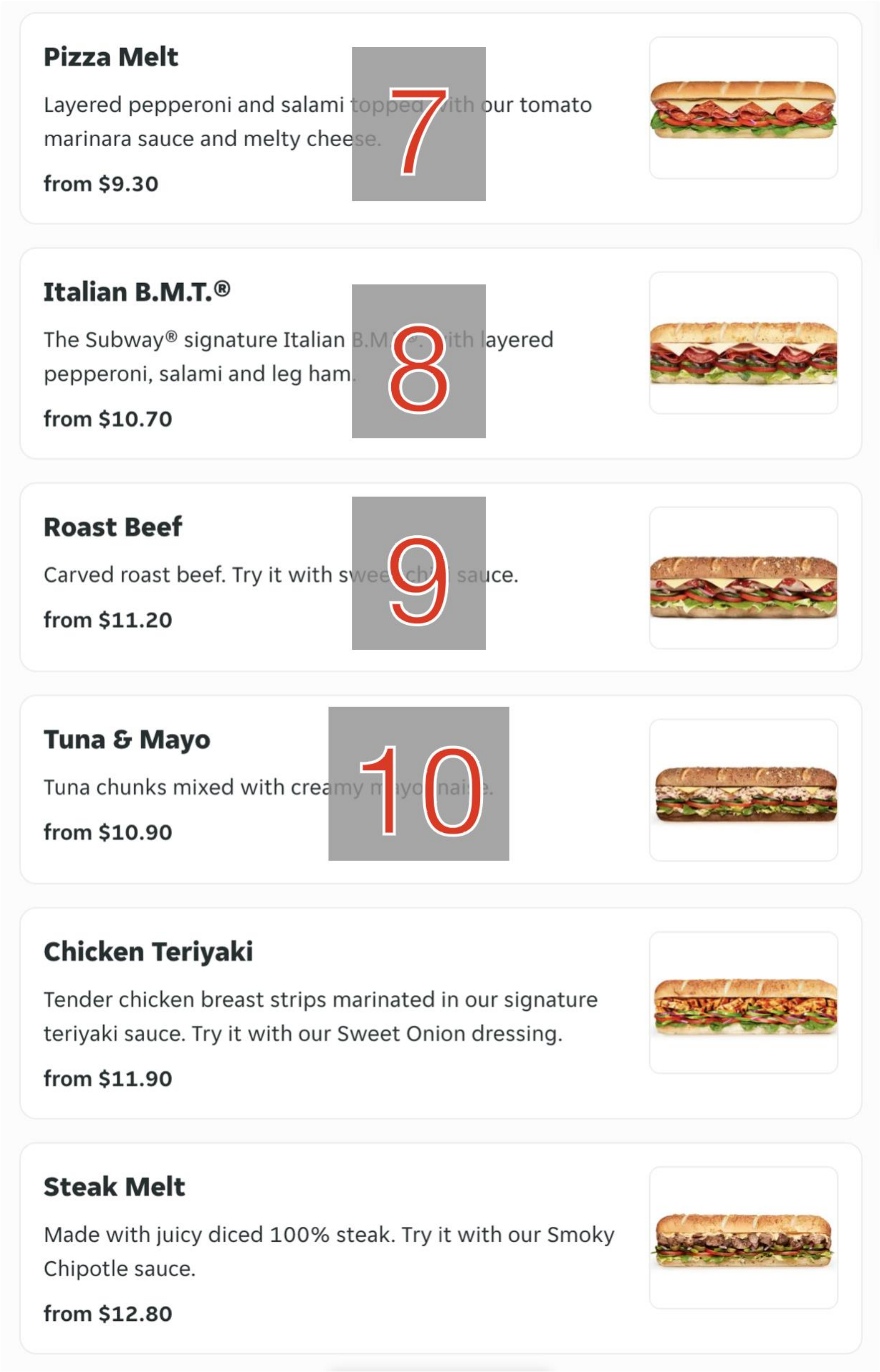

Delivereasy Food Outlets Example

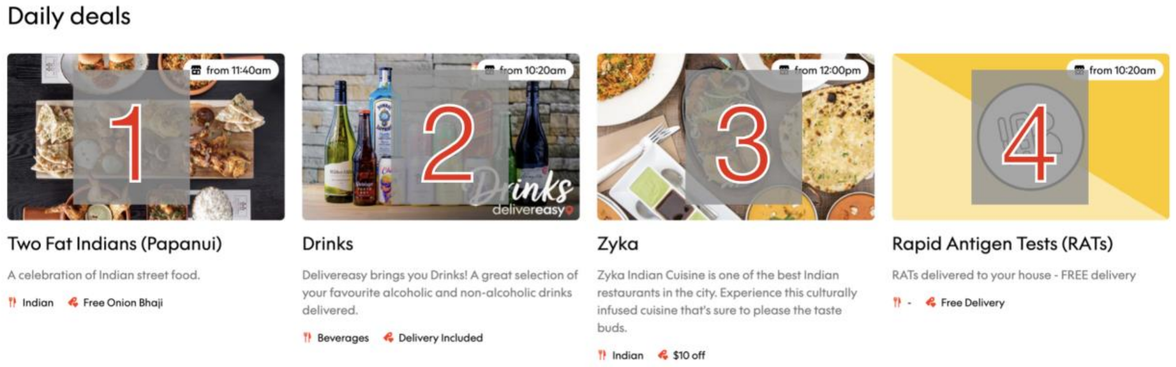

Note the presence of RATs (excluded from analysis), illustrating the continued influence of COVID.

Page scroll...

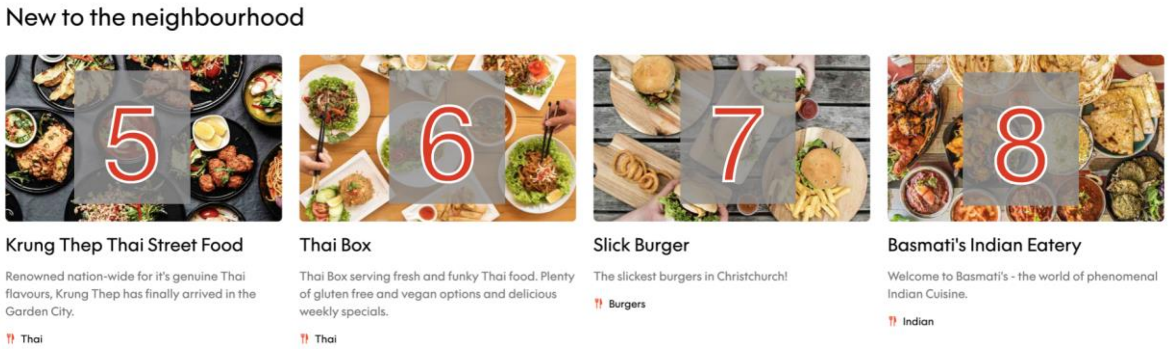

Page scroll...

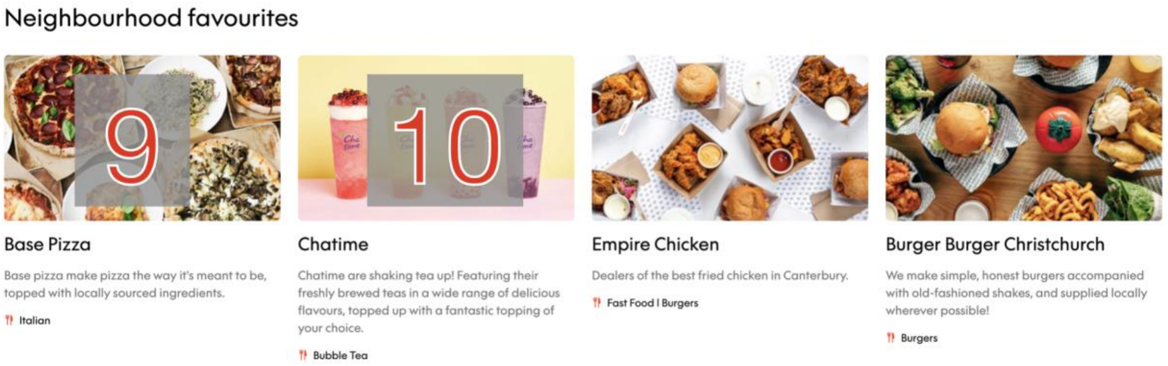

Delivereasy Menu Items Example

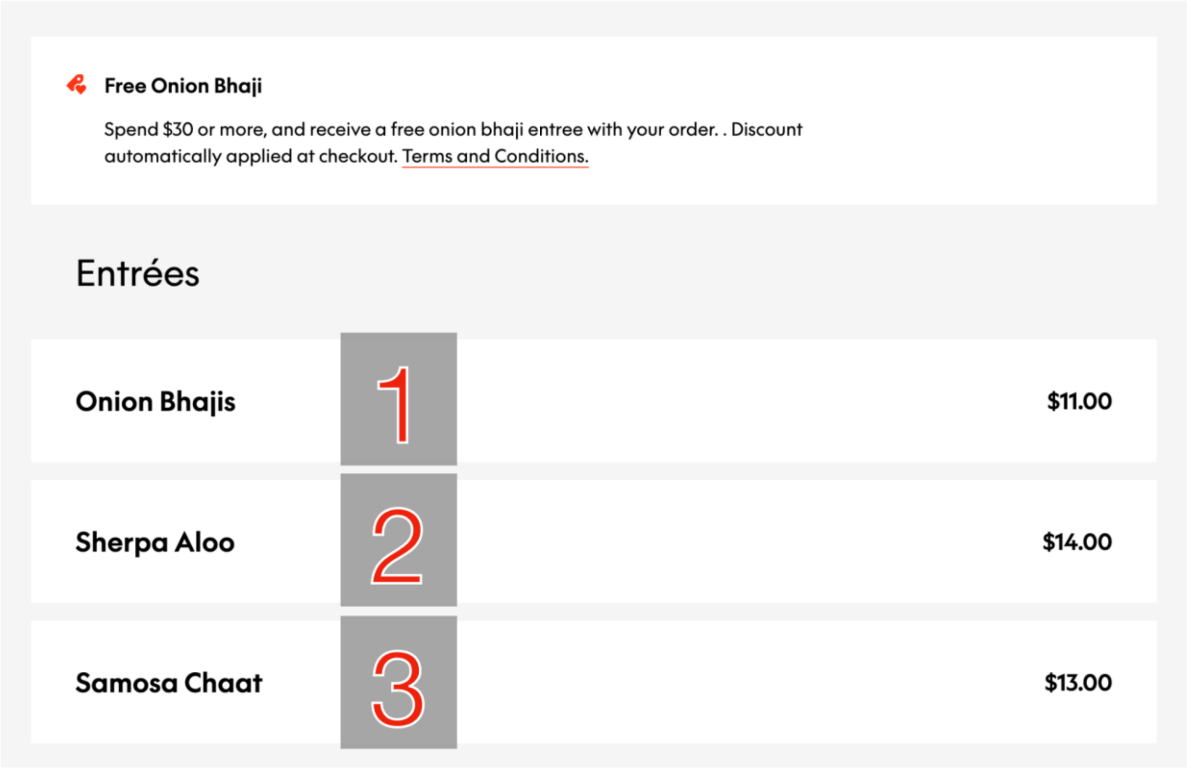

This screenshot illustrates a complication of judging the “first ten” items shown to the user. There is a promotional item above the menu for a food item, which we have not counted as the first menu item, though it is likely to have some effect on the behaviour and consumption of unhealthy foods at the core of our study. This type of advertising may be investigated in a future study, using the set of screenshots collected during the current work.

Delivereasy Menu Items Example (continued)

After page scroll…

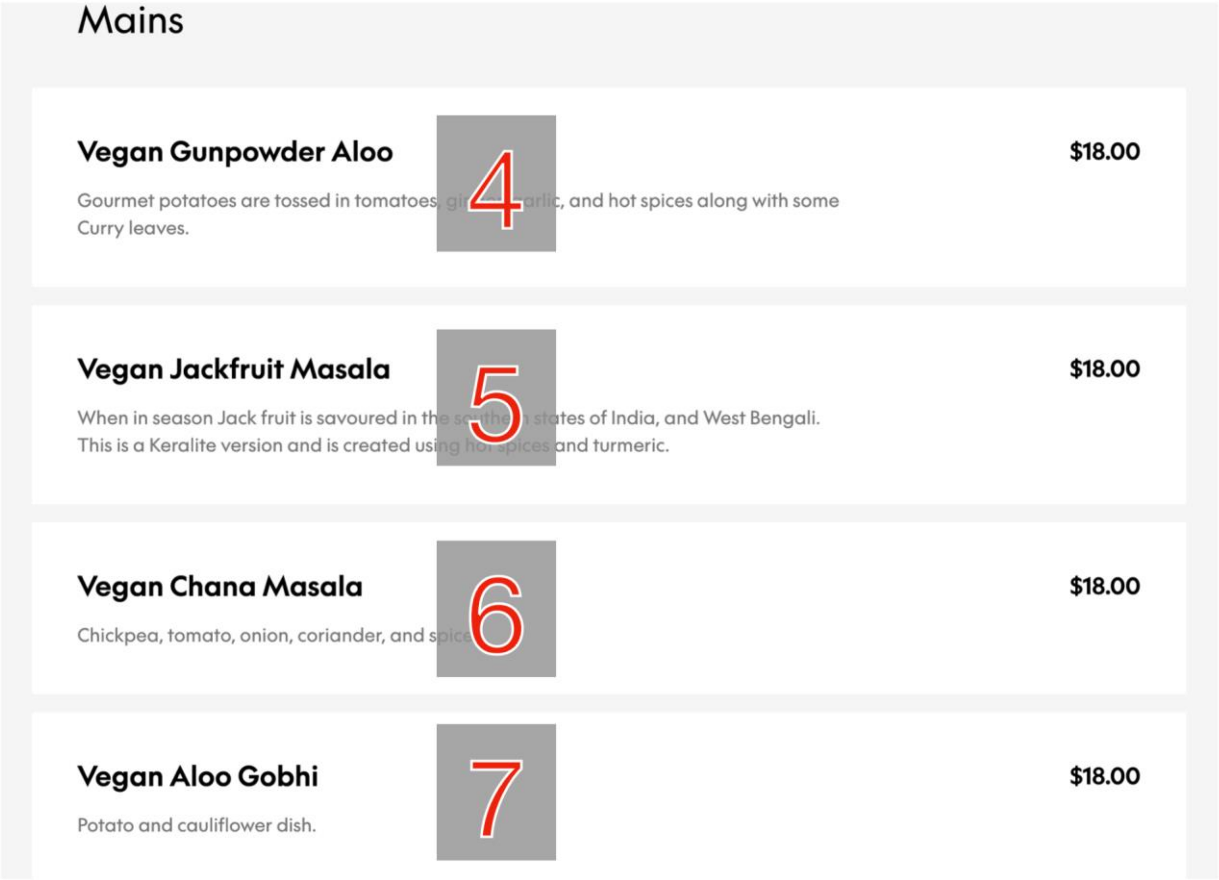

Delivereasy Menu Items Example (continued)

After page scroll...

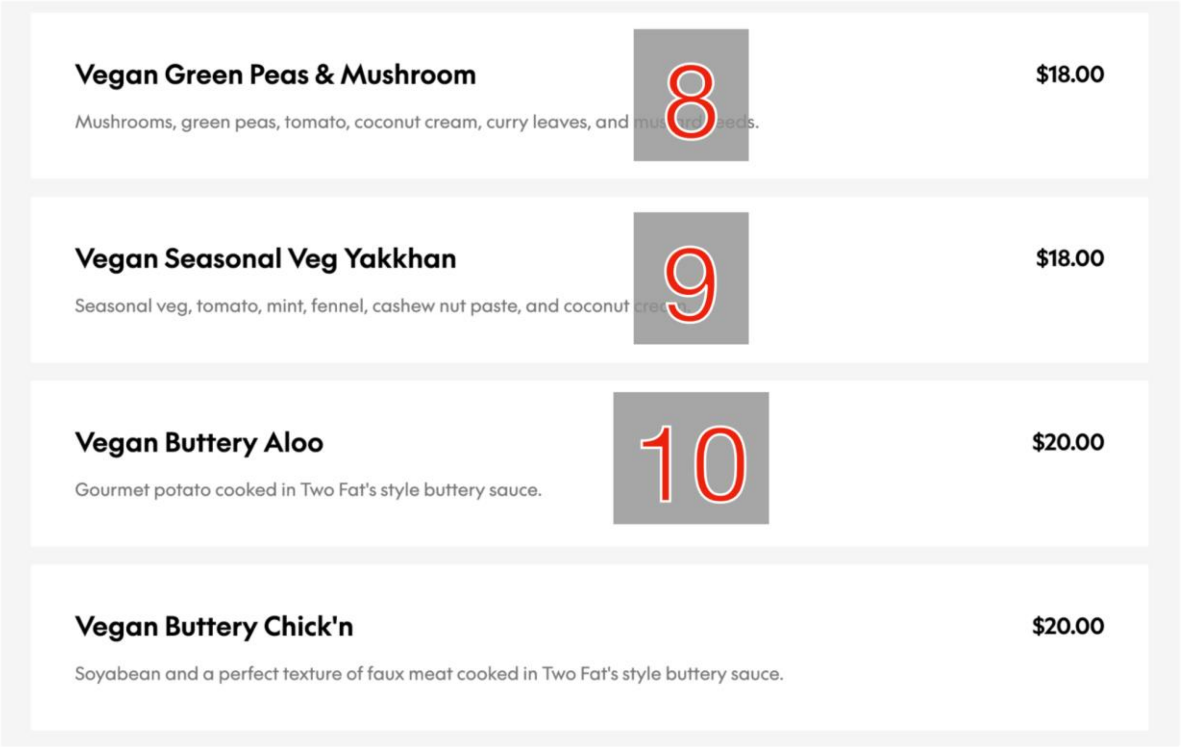

Supplement: Supplementary file 1 [file nutrients-14-04228-s001.zip › nutrients-1939804-supplementary.pdf]
